# The Impact of Biomass Treatment and Plasticizers on the Properties of Chicken Feather-Based Biodegradable Films

**DOI:** 10.3390/polym18080969

**Published:** 2026-04-16

**Authors:** Sarah Montes, Emmi Nuutinen, Julen Vadillo, Alaitz Rekondo, Hans-Jürgen Grande, Jonna Almqvist

**Affiliations:** 1 CIDETEC, Basque Research and Technology Alliance (BRTA), Paseo Miramón 196, 20014 Donostia-San Sebastian, Spain; 2 Sustainable Products and Materials, VTT Technical Research Centre of Finland, Tietotie 2, FI-02044 Espoo, Finland; 3 Advanced Polymers and Materials: Physics, Chemistry and Technology Department, University of the Basque Country (UPV-EHU), Avda. Tolosa 72, 20018 Donostia-San Sebastian, Spain; 4 RISE Research Institutes of Sweden, Department of Biorefinery and Energy, S-892 50 Örnsköldsvik, Sweden

**Keywords:** biodegradable films, feather waste, valorisation, steam explosion, deep eutectic solvent, mechanical grinding

## Abstract

The poultry sector generates large amounts of feather waste every year, providing an abundant keratin-rich residue that is difficult to valorise due to its crosslinked and highly compacted crystalline structure. In the present work, with the aim of promoting its use in biodegradable plastic films, environmentally friendly processes, such as mechanical grinding (compactor grinder, CG), deep eutectic solvents (DES), and steam explosion process (SE) are being explored as alternatives to conventional chemical processes. Thus, biodegradable feather-based films were produced by compounding treated feathers in a torque rheometer at 40 wt.% with glycerol, ethylene glycol, and 1,2-propanediol (propylene glycol), followed by hot pressing. All formulations produced homogeneous and translucent films, which were characterized in terms of colorimetric properties and thermal and mechanical behaviour, as well as their degradation in soil conditions, revealing pronounced differences in properties as a function of the specific combination of feather treatment and plasticizer employed. Interestingly, soil disintegration tests revealed the fastest degradation of films of DES-treated feathers plasticized with glycerol. Overall, controlling feather treatment and plasticizer type enables tuning of mechanical performance and biodegradation, supporting keratin-based films as a viable route for feather waste valorisation.

## 1. Introduction

Globally, the poultry industry is thought to produce roughly 2 million tons of industrial feather waste each year [1]. Although feathers are a valuable source of protein (90% of feathers are constituted of keratin), the management and valorisation of this byproduct are not simple due to their recalcitrant nature [2].

Due to the particular structure of keratin, i.e., extensive crosslinking of disulfide bonds and compact β-sheet packing inside the polypeptide chain, this byproduct is difficult to dissolve and process in conventional solvents. To disrupt the disulfide bonds in keratin, several chemical methods, such as reduction, oxidation, or sulfitolysis, can be applied. However, these processes frequently rely on the use of toxic or non-environmentally friendly chemicals [3].

Alternative approaches for feather treatment include the use of deep eutectic solvents (DES), solutions of Lewis or Brønsted acids and bases which form a eutectic mixture [4], and physical techniques such as steam explosion (SE). The latter reduces the stiffness of the fibre structure and increases the accessibility of the biomass polymer for subsequent processing by hydrolysis or fermentation [5,6]. Moreover, mechanical grinding of feathers has been used as a straightforward method to obtain feather fibres, which can be used in the preparation of nonwoven materials for geotextiles [7,8].

Any of the methods used for feather treatment affect its final characteristics, altering its physical, chemical, or functional properties [9]. Due to their ability to retain the original polymer’s properties as well as their recyclable nature, DES have been utilized as a mild alternative to chemical treatments to extract keratin and other naturally occurring polymers from their original sources [10]. DES features can be tailored to the target polymer to be treated or extracted by selecting the type and hydrogen bonding pair [4]. In recent studies, an aqueous DES was used to convert feathers into homogeneous keratin-based raw material by combining urea, sodium acetate, and small amounts of water. In order to maximize the yield of regenerated keratin, the processing parameters were optimized [11].

Concerning the steam explosion process, it represents another green strategy for protein extraction [5]. The method, widely used in lignocellulosic biomass pretreatment [12], relies on subjecting the biomass to extremely hot steam and allowing steam penetration into compact structures, followed by a rapid and explosive decompression that occurs in milliseconds [6,13]. Steam explosion has been used to disrupt disulfide bonds in feather fibres using only water [14]. Due to the large thermal and mechanical separation effect, the produced material mainly revealed the existence of shapeless particles with a wide particle size distribution. The extraction process can be improved by the use of alkali solutions [15], but in the case of feather keratin, these conditions result in a serious degradation of the peptide chain [16,17], which limits the suitability of the resulting keratin for specific applications.

Regardless of the process used for the treatment of feathers and the extraction of keratin, several valorisation routes have been explored to obtain materials that may benefit from the inherent properties of feather keratin, such as biocompatibility and biodegradability. In this sense, for biotechnological applications, feather keratin can be transformed into a variety of forms that include films, sponges, and fibres, or be blended with other polymers [18,19,20,21,22,23].

One of the most promising valorisation routes for feathers, beyond the field of biotechnology, is the preparation of bioplastics (according to the standard UNE-CEN/TR 15932 [24], the term bioplastic encompasses materials that are bio-based, biodegradable, or both), as it offers the possibility of using large quantities of feathers [25,26,27,28]. Bioplastics derived from feather keratin can be made through different approaches, including the use of plasticizers in combination with reducing agents and blended with other polymers [27,29,30]. In most cases, the application of a pretreatment to feathers is required to enhance the biodegradability of the resulting material. Despite its being an intrinsically biodegradable biopolymer, the biodegradation rate of feather keratin is slower compared to that of cellulose or other biodegradable polymers, so the application of a pretreatment may be of significant relevance in accelerating the biodegradation rate in certain environments, such as soil biodegradation [9]. In this context, the development of keratin-based biodegradable films holds significant potential for applications such as the production of mulch films for crop protection, which, after use, can biodegrade and disintegrate under soil conditions.

In the present work, biodegradable feather-based films were prepared by blending treated feathers with glycerol, ethylene glycol and 1,2-propanediol plasticizers. For this purpose, treated feathers were produced by applying different methods: mechanical grinding using a compactor grinder (CG), DES treatment and the SE process. The effect of the feather treatment and the type of plasticizer on the mechanical and thermal properties and the influence on the degradation of films in soil were investigated. To the best of our knowledge, the combined effect of feather treatment and plasticizer type on the properties of hot-pressed keratin-based films has not yet been systematically compared.

Faster disintegration of the films was anticipated due to the incorporation of biodegradable plasticizers and the inclusion of treated feathers. Films derived from feather keratin may represent a sustainable valorisation pathway for feather waste, simultaneously contributing to waste reduction and the development of biodegradable materials capable of replacing conventional plastics.

## 2. Materials and Methods

### 2.1. Materials

Sanitized feathers were supplied by Grupo SADA (Madrid, Spain). Feathers were washed at 95 °C for 2 h with an alkaline soap solution and dried at 60 °C for 24 h. Sterilization was performed at 126 °C for 30 min with pressurized steam treatment. The absence of pathogens was confirmed by microbiological detection.

The 99.0–100.5% urea was purchased from Sigma-Aldrich (Steinheim, Germany), >99% sodium acetate anhydrous from Sigma-Aldrich (St. Louis, MO, USA), 99.5% glycerol (boiling point 290.0 °C) from Scharlau (Barcelona, Spain), extra pure ethylene glycol (boiling point 197.3 °C) from Scharlau (Spain) and 1,2-propanediol (propylene glycol, boiling point 188.2 °C), >99.5%, from Sigma-Aldrich (Saint-Quentin-Fallavier, France).

### 2.2. Feather Treatment

#### 2.2.1. Mechanical Grinding by Compactor Grinder (CG)

Sanitized feathers were mechanically ground into 2–15 mm pieces using an E-compactor (VTT, Espoo, Finland) in which the feathers are pressed through a die using pan grinder rollers.

#### 2.2.2. Steam Explosion (SE) Treatment

Raw feathers were placed in a 40 L custom-built batch reactor with a capacity of 4 kg of feathers/batch. Based on previous experiments’ results, water steam at 160 °C and 0.62 MPa was introduced into the reactor for 2 min. After that, within <1 s the pressure was reduced to atmospheric pressure. The structure of the feathers, including some disulphide bonds, was disrupted by the pressure change.

Steam-exploded feathers were dried in an oven at 105 °C for 8 h and then ground with a hammer mill (Hosokawa 100 UPZ, plate beater, Hosokawa Alpine Aktiengesellschaft, Augsburg, Germany) using a sieve insert size of 0.5 mm.

#### 2.2.3. Deep Eutectic Solvent (DES) Processing

DES processing was carried out following the procedure previously described [11]. In brief, using a 15 L closed reactor equipped with a mixer, roughly ground feathers (8 wt.%) were added to preheated, clear, freshly prepared (70 °C) solvent consisting of NaOAc and urea (molar ratio of 1:3) and with a small addition of water (10 wt.%). The dissolution was carried out at 95 °C for 7 h. After the dissolution, the solution was poured into water (20 L), in which part of the dissolved feather keratin regenerated (~50%) and part (~40%) was dissolved in water together with the DES components. Undissolved feathers (~10%) were mixed together with regenerated keratin. The solid fraction (regenerated keratin and undissolved feathers) was separated using a Büchner funnel (60 mm), washed with water, freeze-dried and further used in this study. The process yield was calculated based on the weight of regenerated keratin relative to the initial amount of feathers.

### 2.3. Blend Preparation

Previously dried (overnight in a vacuum oven at 100 °C) compactor grinder-treated feathers, steam explosion-treated feathers and DES-processed feathers were first manually mixed with different plasticizers in a mass ratio of 3:2. Blends were then prepared using the torque rheometer HAAKE PolyLab QC (Thermo Fisher Scientific, Karlsruhe, Germany) at 150 °C. The mixture was added to the torque rheometer stepwise within 10 min using 30 rpm mixing speed. Once all the material was added to the torque rheometer, the mixing speed was increased to 70 rpm for 5 min. Table 1 shows all the blends prepared combining each type of treated feathers (CG-, DES- and SE-treated) with the three types of plasticizers.

### 2.4. Bioplastic Film Preparation

Films were obtained by compression moulding of blends using a Vogt 600T laboratory hot press machine (Maschinen + Technik Vogt GmbH, Möhnesee, Germany). The pressing was done at 150 °C for 5 min and under 200 bar pressure. Before pressing, the material was placed inside square frames (12 cm × 12 cm × 0.55 cm or 5 cm × 5 cm × 0.2 cm; length × width × thickness).

### 2.5. Characterization Techniques

#### 2.5.1. Field-Emission Scanning Electron Microscopy (FE-SEM)

The morphology of the treated feathers was determined by using an Ultra Plus field-emission scanning electron microscope (FE-SEM), (Carl Zeiss Microimaging, Oberkochen, Germany). All samples were initially coated with a thin layer of gold (Au). This coating process was performed to enhance the conductivity of the samples, thereby ensuring optimal imaging quality during the FE-SEM examination.

#### 2.5.2. X-Ray Diffraction (XRD)

The influence of the different treatments on feather crystallinity was evaluated by X-ray diffraction (XRD). Measurements were performed using a D8 Discover diffractometer (Cu Kα radiation, λ = 0.154 nm, Bruker, Billerica, MA, USA) equipped with a LynxEye PSD detector (Bruker AXS GmbH, Karlsruhe, Germany). Diffractograms were collected over a 2θ range of 5–80°, at a scanning rate of 0.03°/s.

The relative crystallinity of the feathers was quantified through deconvolution of the diffraction peaks. The diffractograms were first fitted using the Lorentzian function, which is the most appropriate model for this type of material [30]. The relative crystallinity of each sample was then calculated from the ratio between the integrated areas of the crystalline and amorphous components.

#### 2.5.3. Colorimetry

The CIELAB colour coordinates were determined using a CM-2600d spectrophotometer (Konica Minolta, Tokyo, Japan) with a d/8° geometry under SCE (Specular Component Excluded) conditions, in order to eliminate the contribution of the specular reflection and to evaluate the apparent colour of the translucent films. The measurements were made with CIE standard illuminant D65 and a 10° standard observer. Three measurements were made per material, and the average and standard deviation of the L*, a* and b* parameters were calculated for every formulation.

#### 2.5.4. Thermogravimetric Analysis

The thermal stability of the films was measured by thermogravimetric analysis using a TGA Q500 (TA Instruments, New Castle, DE, USA). Dynamic measurements were performed from 25 to 600 °C at a heating rate of 10 °C/min by using a constant nitrogen flow of 60 mL/min.

#### 2.5.5. Differential Scanning Calorimetry

Thermal properties of plasticized samples were determined with a Discovery DSC 25 auto (TA Instruments, New Castle, DE, USA) at a scan rate of 10 °C/min. Samples were first heated from room temperature to 120 °C and kept there for 5 min to remove the moisture. Then samples were cooled down to 20 °C, and DSC analysis was then carried out over the temperature range 20–250 °C. The measurements were carried out under a nitrogen atmosphere (50 mL/min).

#### 2.5.6. Mechanical Testing

The tensile strength and elongation at break were determined using a universal testing machine model 3365 (Instron, Norwood, MA, USA) equipped with a 5 kN load cell. Tests were performed at a crosshead speed of 50 mm/min, with an initial gauge length of 50 mm, and controlled by Bluehill Lite software (version 2.21.748) developed by Instron (Norwood, MA, USA). Average tensile strength (σ_b_), elongation at break (ε_b_) and elastic modulus (E) were calculated from the resulting stress–strain curves. The measurements were done at 23 °C and 50% RH. The average of ten specimens with an effective length of 7 cm, a width of 1.5 cm and a thickness of 1 mm from each sample was reported.

#### 2.5.7. Film Degradation Testing

Bioplastic films were cut into 30 mm × 40 mm rectangular specimens for testing. The degradation was determined by measuring the weight loss of the specimens buried in a mature compost soil (elemental composition of compost soil: 23.14% C, 2.95% H, 2.97% N) for 12 weeks. Each specimen was buried in the compost soil and incubated at a temperature of 25 ± 2 °C. The compost moisture (50 ± 10%) was kept constant by the addition of water. Each specimen sample was weighed once a week. The weight loss $W_{loss}$ (%) was calculated using Equation (1):

(1) $$W_{loss}(\%)=\frac{W_{initial}-W_{final}}{W_{initial}}\times 100$$

where $W_{initial}$ and $W_{final}$ are the weights of the specimen before and after being buried in compost soil.

## 3. Results and Discussion

### 3.1. Effect of Treatments on Feather Morphology and Structure

#### 3.1.1. Morphology

**Figure 1:**
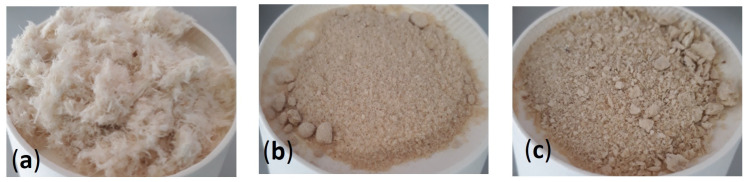
Treated feathers after (**a**) compactor grinder treatment, (**b**) SE treatment and (**c**) DES treatment.

The different types of feathers obtained from each treatment are shown in Figure 1. Treated feathers obtained via CG exhibit a predominantly fibrous morphology, whereas material produced by SE or DESs displays a particulate morphology with slight colouration.

**Figure 2:**
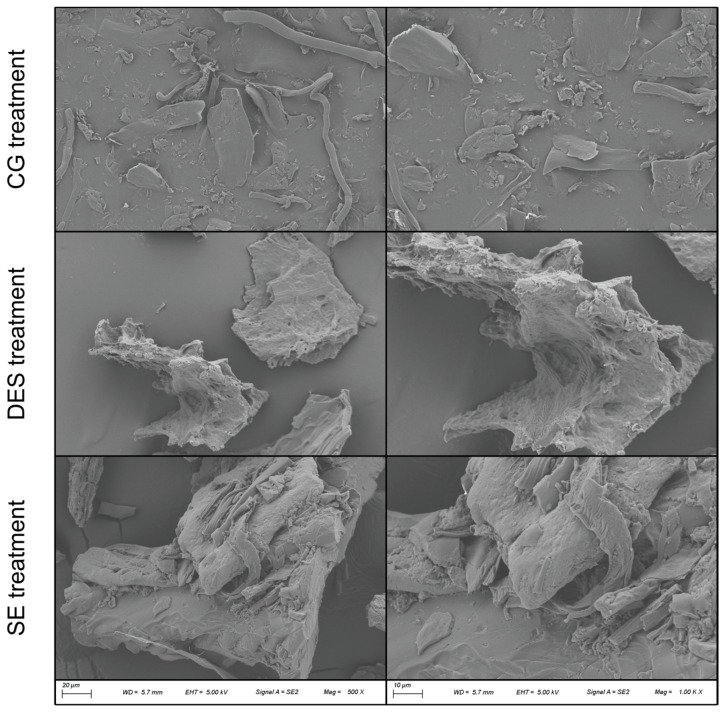
FE-SEM micrographs of feather keratin obtained with the three treatments: magnification 500× (**left**) and 1000× (**right**).

In the higher-magnification FE-SEM micrographs (Figure 2), the same trends are observed. Material produced by CG retains features of the native feather architecture, including residual quills and small feather fragments. In contrast, DES-treated samples consist predominantly of irregular particulates exhibiting a highly porous surface. SE treatment yields particles with markedly increased surface roughness, exhibiting pronounced surface asperities. This modification is consistent with mechanical tearing delamination of the feather matrix during SE processing [9].

#### 3.1.2. Crystallinity

**Figure 3:**
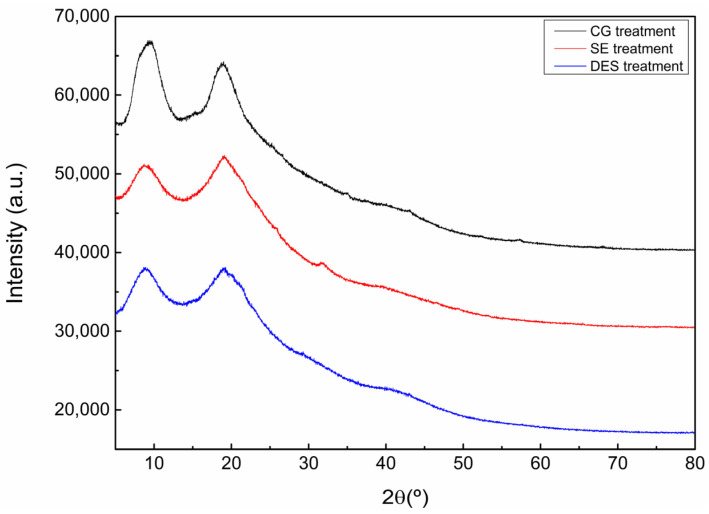
XRD diffractograms of differently treated feathers.

Figure 3 presents the X-ray diffraction patterns of CG-, SE- and DES-treated feathers. The diffractograms exhibit two broad characteristic peaks located at approximately 9° and 19° (2θ). The peak near 9° is associated with the α-helix structure, while the one around 19° corresponds to the β-sheet conformation [31]. Comparing the curves obtained for the different treatments, neither apparition of signals nor displacement of the existing ones was observed, with all materials showing a similar diffractogram.

The relative crystallinity of each sample was determined through the deconvolution of the obtained diffractograms. The results are summarized in Table 2. The results revealed a decrease in relative crystallinity for both SE and DES treatments compared to the CG one. In the case of the SE treatment, the applied temperature and pressure contributed to the disruption of the crystalline network, as reported in other studies involving different types of organic materials [32,33]. Moreover, for the DES treatment, similar relative crystallinity values were observed, suggesting that the interaction of the DES components with the polymer chains may have hindered the reorganization of the crystalline regions during treatment.

### 3.2. Film Characterization

#### 3.2.1. Colorimetry in CIELAB (L*a*b*)

The CIELAB colour coordinates were measured under SCE conditions to evaluate the apparent colour of the translucent films. Table 3 summarizes the obtained L*, a*, and b* values for the different treatments and plasticizers. These parameters provide quantitative insight into the variations in lightness and chromaticity associated with each processing method and formulation.

The CIELAB colour coordinates obtained under SCE conditions showed significant variations depending on both the treatment applied to the feather keratin and the type of plasticizer used. Films prepared with the CG fraction were the lightest (L* ≈ 58) and exhibited yellowish-brown hues (b* ≈ 47), while those derived from SE treatment were notably darker (L* ≈ 45) and redder (a* ≈ 17), indicating a higher degree of colouration. The DES-based materials presented intermediate colour values, suggesting a milder modification during processing. Within each treatment, replacing polypropylene glycol with ethylene glycol or glycerol generally decreased lightness and increased the red component. This trend may be related to the higher hydroxyl group density of EG and glycerol, which can modify plasticizer–keratin hydrogen bonding, polarity, and molecular mobility during processing, thereby favouring the formation of coloured compounds through thermally induced non-enzymatic browning pathways [34,35]. Similar effects of polyols on protein matrices and browning-related chemistry have been reported in the literature [36].

#### 3.2.2. Thermal Properties

##### Thermal Stability

**Figure 4:**
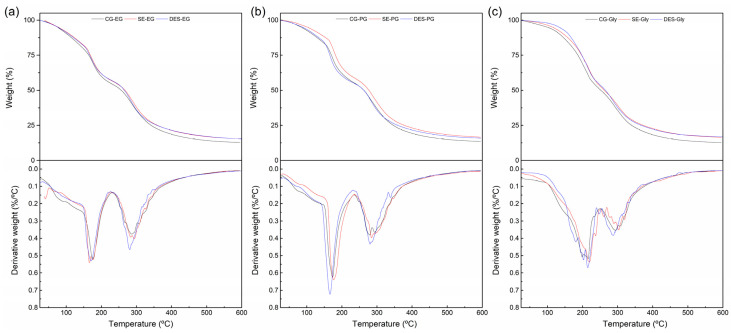
TG and DTG curves for the comparison of feather treatments; plasticized films made from (**a**) ethylene glycol, (**b**) 1,2-propanediol, and (**c**) glycerol.

Figure 4 shows the TG and DTG curves obtained from the different plasticized films. Regarding the thermal stability, no significant differences were observed between the feather treatments or the types of plasticizers used. In all cases, the degradation of each plasticized film consisted of three weight loss steps: an initial gradual weight loss below 150 °C associated with moisture evaporation, a second weight loss between 150 °C and 250 °C attributed to the plasticizer evaporation and a final weight loss beyond 250 °C assigned to the decomposition of the keratin [30].

**Figure 5:**
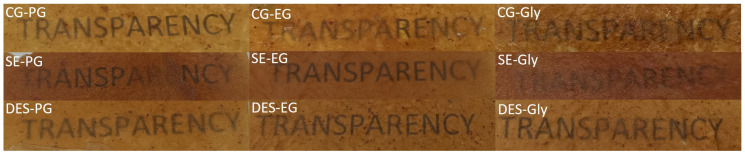
Films obtained from the combinations of plasticizers and treated feathers.

It is worth noting that during the processing of feather-based materials with each of the plasticizers in the torque rheometer, a progressive darkening of the material was observed (Figure 5). The chemical origin of this change was not specifically investigated in this study. According to the literature, the discolouration during protein processing may be attributed to a combination of thermochemical processes, including the denaturation and oxidation of amino acids present in keratin (notably aromatic residues such as tyrosine and tryptophan), as well as the thermal breakdown of disulfide bonds [37]. Additionally, the plasticizers used can undergo partial thermal degradation at elevated temperatures, generating carbonyl compounds capable of reacting with amino groups in the protein matrix, thus promoting condensation reactions. This protein–plasticizer interaction may explain the formation of pigmented compounds, which contribute to the browning of the final material [38,39].

##### Effects of Plasticizers on Thermal Properties

**Figure 6:**
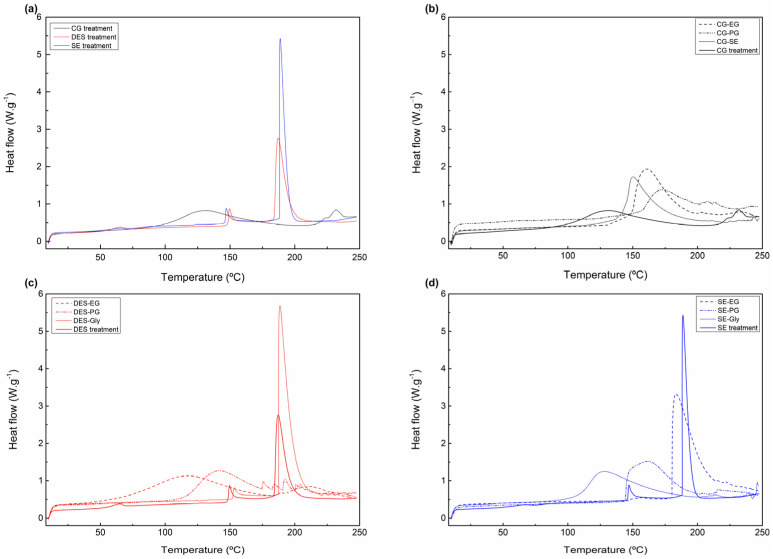
DSC curves of (**a**) feather treatment comparison andplasticized films made from (**b**) compactor-grinder-, (**c**) DES- and (**d**) steam explosion- treated feathers.

The thermal transitions of the three types of treated feathers and their respective plasticized films were investigated by DSC. Representative heat flow curves are shown in Figure 6.

CG-treated feathers exhibited two endothermic transitions in a similar way to raw feathers: a broad peak below 150 °C attributed to the evaporation of residual moisture and a sharp narrow peak at around 233 °C that might be due to the melting of the crystalline region [27]. In the case of DES and SE treatments, two endothermic peaks are also observed. A small and sharp peak appears around 150 °C, commonly attributed to the thermal denaturation of keratin under partially hydrated conditions or the loss of structural water after treatments. These treatments modify the protein matrix, increasing the accessibility of residual water or disrupting the secondary structure of keratin [40]. A second, more intense and narrower peak is observed at around 190 °C. This peak might be attributed to the onset of the thermal degradation of keratin, specifically to the breakage of structural bonds (mainly hydrogen bonds and disulfide interactions) that are altered or partially broken by chemical and physical treatments [40,41].

Regarding plasticized films, two main thermal transitions can also be observed in most of the samples: a broad endothermic peak corresponding to the gradual plasticizer and moisture evaporation at temperatures between 100 °C and 210 °C approximately and a small endothermic peak below 250 °C, attributed to crystalline melting of the keratin. However, this peak is not present in all the thermograms, as some samples begin to degrade at temperatures above 200 °C.

With respect to the influence of the plasticizer type utilized, glycerol appears to induce the most pronounced effects on the thermal behaviour of the samples in all treatments (SE, DES, CG), with the highest increase in the heat flow at elevated temperatures. This could be attributed to its higher molecular weight and more complex interactions with the treated biomass, enhancing plasticization. For the plasticizer ethylene glycol (EG), the films with SE-treated feathers show a much broader endothermic peak with a lower intensity. This might indicate weaker plasticizing effects compared to glycerol. Finally, propylene glycol (PG) seems to yield more stable profiles, particularly in the combination with compactor grinder-treated feathers. The thermal effects observed with PG are less pronounced than with GLY, suggesting that PG is a less effective plasticizer for the biomass under these specific treatment conditions [42].

#### 3.2.3. Mechanical Properties

**Figure 7:**
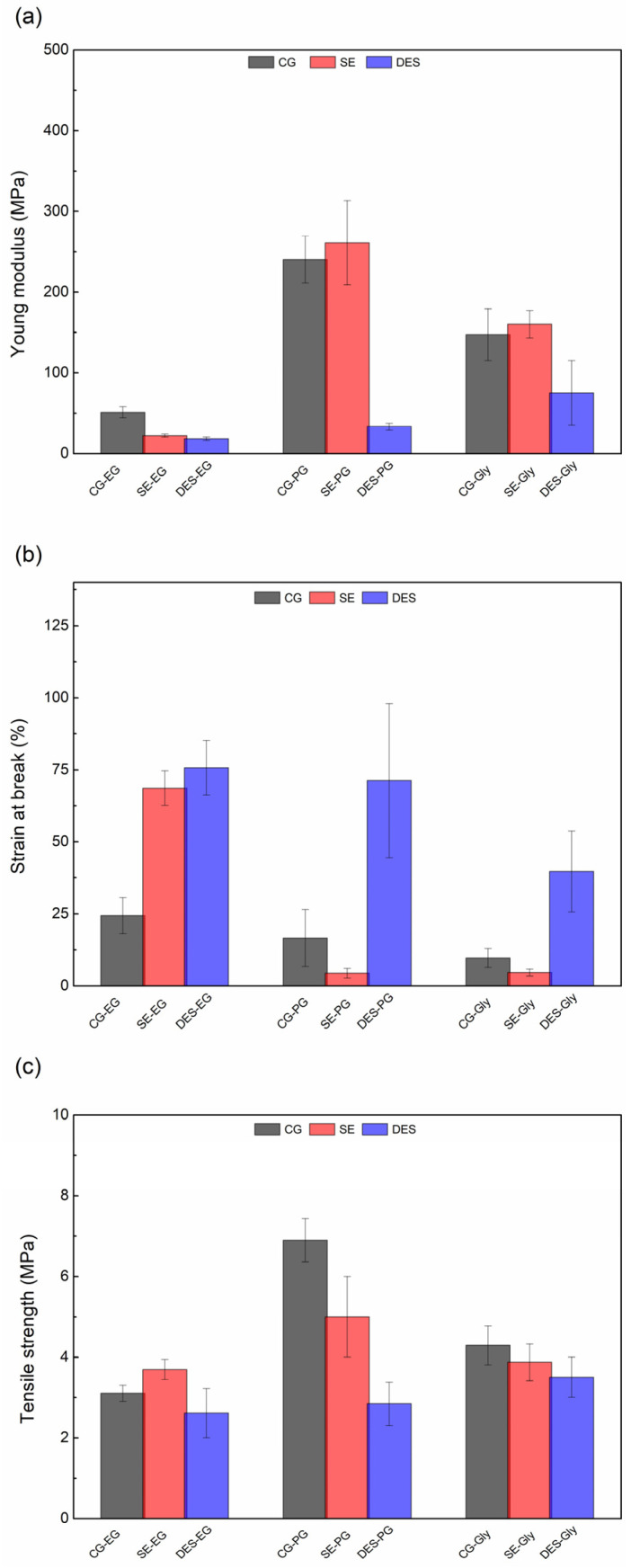
(**a**) Young’s modulus, (**b**) elongation at break and (**c**) tensile strength values of differently treated feathers plasticized with glycerol, ethylene glycol and propylene glycol.

The mechanical performance of feather-derived materials plasticized with ethylene glycol (EG), propylene glycol (PG), or glycerol (Gly) was quantified under uniaxial tension in terms of tensile strength (σ), elongation at break (ε), and Young’s modulus (E). Figure 7 summarizes the results, revealing pronounced variation in mechanical response as a function of both the feather pretreatment (CG, SE, DES) and the plasticizer identity.

In the case of CG treatment, the combination of mechanically treated feathers with propylene glycol (PG-CG) presented the highest rigidity (240 MPa) and tensile strength (6.9 MPa) among all formulations with this treatment, while the highest elongation at break was obtained using ethylene glycol (EG-CG). Similarly, steam explosion (SE) treatment combined with propylene glycol (PG-SE) achieved the highest Young’s modulus value of the entire study (261 MPa) and the highest tensile strength among the different plasticizers used with SE treatment (5 MPa), while ethylene glycol (EG-SE) produced highly flexible materials with 68.6% elongation at break. This variation suggests that PG forms stronger interactions with the keratin structure in both cases, possibly due to its molecular size and hydroxyl group positioning, which favour intermolecular hydrogen bonding [27,41,43]. The comparatively better mechanical performance of PG-plasticized films, despite its lower plasticizing efficiency, may arise from a balance between its molecular size, polarity, and ability to interact with the polymer matrix through hydrogen bonding. These factors can modulate intermolecular interactions and chain mobility, and it has been reported that molecular weight and polarity strongly influence polymer dynamics and the strength of interfacial interactions, which in turn affect mechanical behaviour [44].

Finally, DES treatment produced materials with pronounced flexibility regardless of the plasticizer used. The combination EG-DES achieved the maximum elongation at break in the study (76.7%), while PG-DES also showed high flexibility (71.2%) but with a dramatic reduction in rigidity compared to other treatments. The observed reduction in stiffness may be attributed to the reduction in crystallinity reported after DES processing, together with DES-induced changes in the surface morphology and heterogeneity of the feather-derived particles and films [45]. The uniform flexible behaviour suggests that DES treatment fundamentally disrupts the keratin crystalline domains and intermolecular interactions, resulting in a more amorphous and accessible matrix for plasticization [11].

These results suggest that the observed differences in mechanical properties arise from specific protein–plasticizer interactions in each combination. Feather keratin is primarily composed of crystalline β-sheet structures stabilized by disulfide bonds [46,47]. The different treatments may specifically modify these structures: CG does not significantly alter the keratin structure and shows the greatest crystallinity, yielding maximal tensile strength; SE treatment primarily removes the amorphous regions, producing materials with high stiffness; and DES treatment disrupts both intermolecular interactions and crystalline domains of the protein matrix, resulting in a more amorphous structure and thus films with the highest elongation at break, attributed to the easier penetration of plasticizers.

#### 3.2.4. Degradation of the Bioplastic Films

**Figure 8:**
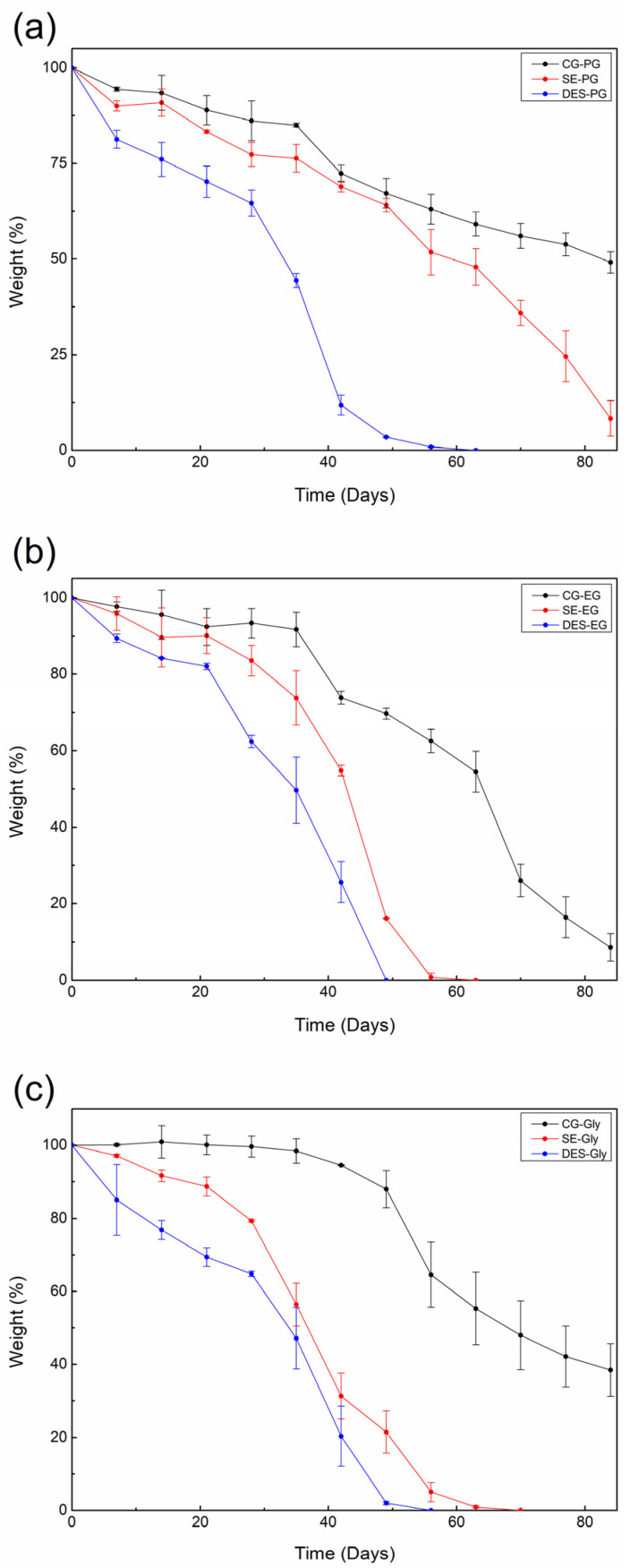
Weight loss in soil of (**a**) propylene glycol-, (**b**) ethylene glycol- and (**c**) glycerol-plasticized films.

**Figure 9:**
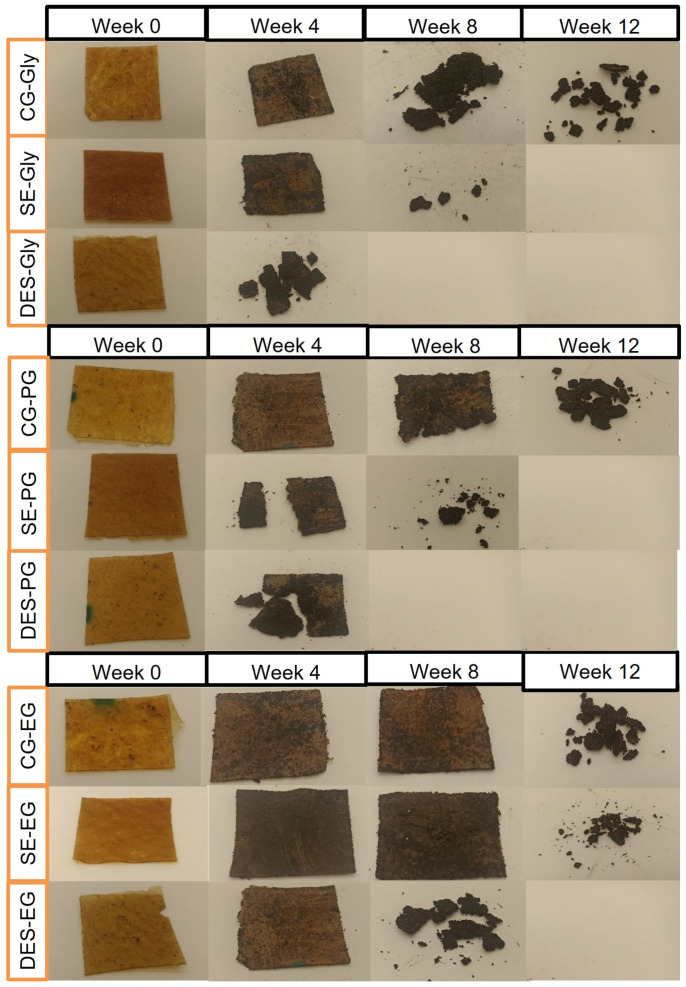
Monitoring of film disintegration under experimental conditions.

The degradation of the films was measured through the disintegration of the materials and monitored weekly by measuring weight loss and performing optical assessments. Films produced from treated feathers combined with the different plasticizers showed significantly different levels of soil disintegration, as summarized in Figure 8 and Figure 9.

Across all combinations, the films from feathers treated with DES exhibited the fastest and most complete weight loss, reaching near-complete disintegration within approximately 40–50 days, followed by those treated with SE, with the majority of samples degrading in between 40 and 70 days, while the CG films displayed the slowest degradation, retaining over 50% of their initial weight after 80 days, regardless of the plasticizer used.

These findings indicate that DES pretreatment substantially facilitates soil disintegration of feather-based films due to enhanced protein denaturation and increased exposure of hydrolysable functional groups. This treatment disrupts keratin structure, thereby increasing both hydrophilicity and enzymatic accessibility, thus accelerating microbial colonization and degradation. In contrast, SE treatment only partially hydrolyses keratin, resulting in moderate biodegradation, whereas CG provides minimal structural changes, limiting microbial attack [48,49,50].

Plasticizer choice also plays a key role: glycerol-based samples degraded faster than those plasticized with propylene glycol or ethylene glycol, especially when combined with DES or SE treatments. Glycerol facilitates higher biodegradability, likely due to its strong interaction with protein matrices, which increases film flexibility and water uptake, further promoting microbial access. Ethylene glycol and propylene glycol also enhance disintegration relative to non-plasticized controls, but to a lesser degree than glycerol [51].

## 4. Conclusions

In the present work, feathers treated by compactor grinding (CG), deep eutectic solvents (DES), and steam explosion (SE) were successfully processed with different plasticizers (propylene glycol, ethylene glycol, and glycerol) to obtain homogeneous and translucent keratin-based films. SE and DES treatments reduced keratin crystallinity compared to CG, indicating disruption of the native structure.

Film darkening during processing was attributed to thermochemically induced reactions within the keratin matrix. Despite these changes, all films exhibited similar thermal stability. DSC results confirmed that CG preserved native-like crystallinity, while DES and SE treatments promoted a more amorphous structure.

Mechanical properties were strongly dependent on both treatment and plasticizer: CG and SE combined with propylene glycol produced stiffer and stronger materials, whereas DES with ethylene glycol yielded more flexible films with higher elongation at break.

The highest soil disintegration rates were observed for DES-treated feathers plasticized with glycerol. These results agree with the literature reporting that DES treatments enhance the biodegradability of biomaterials by disrupting crystalline structures and supporting microbial growth, while other studied treatments resulted in lower disintegration rates due to their less disrupting effect on the feather structure.

Considering these results, the selection of the feather treatment and plasticizer should be application-driven and tailored to the targeted final performance. The production of biodegradable keratin-based films may represent a promising pathway for the valorisation of this biomass waste from chicken feathers.

## Figures and Tables

**Table 1:** Blends prepared from treated feathers with different plasticizers.

Reference	Feather Treatment	Plasticizer Type
CG-PG SE-PG DES-PG	Compactor grinder [CG] Steam explosion [SE] Deep eutectic solvent [DES]	Propylene glycol [PG]
CG-EG SE-EG DES-EG	Compactor grinder [CG] Steam explosion [SE] Deep eutectic solvent [DES]	Ethylene glycol [EG]
CG-Gly SE-Gly DES-Gly	Compactor grinder [CG] Steam explosion [SE] Deep eutectic solvent [DES]	Glycerol [Gly]

**Table 2:** Determined relative crystallinity of feather treatments.

Sample	Relative Crystallinity (%)
CG treatment	0.62 ± 0.01
SE treatment	0.55 ± 0.02
DES treatment	0.56 ± 0.02

**Table 3:** CIELAB colour coordinates of prepared films.

Material	Colour Coordinates		
	L*	a*	b*
CG-PG	62.2 ± 0.2	7.1 ± 0.3	48.3 ± 0.1
CG-EG	56.6 ± 1.2	11.6 ± 1.3	48.8 ± 0.7
CG-Gly	56.3 ± 0.5	13.1 ± 1.2	45.01 ± 1.4
SE-PG	45.8 ± 0.5	17.3 ± 0.1	35.6 ± 0.7
SE-EG	46.4 ± 1.7	15.9 ± 0.7	40.1 ± 1.2
SE-Gly	41.9 ± 0.3	17.6 ± 0.5	31.8 ± 1.5
DES-PG	53.8 ± 0.4	12.1 ± 0.7	43.8 ± 0.2
DES-EG	51.6 ± 1.2	10.1 ± 0.4	39.1 ± 1.1
DES-Gly	53.1 ± 0.8	9.9 ± 0.7	40.2 ± 0.4

## Data Availability

The original contributions presented in this study are included in the article. Further inquiries can be directed to the corresponding author.
